# Advancing Emotionally Aware Child–Robot Interaction with Biophysical Data and Insight-Driven Affective Computing

**DOI:** 10.3390/s25041161

**Published:** 2025-02-14

**Authors:** Diego Resende Faria, Amie Louise Godkin, Pedro Paulo da Silva Ayrosa

**Affiliations:** 1 School of Physics, Engineering and Computer Science, University of Hertfordshire, Hatfield AL10 9AB, UK; 2 Turning Point, Leicestershire LE1 3QL, UK; amie.godkin@turning-point.co.uk; 3 LABTED/NEAD and Department of Computer Science, State University of Londrina, Londrina 86057-970, Brazil; ayrosa@uel.br

**Keywords:** emotion-aware technology, affective computing, child–robot interaction

## Abstract

This paper investigates the integration of affective computing techniques using biophysical data to advance emotionally aware machines and enhance child–robot interaction (CRI). By leveraging interdisciplinary insights from neuroscience, psychology, and artificial intelligence, the study focuses on creating adaptive, emotion-aware systems capable of dynamically recognizing and responding to human emotional states. Through a real-world CRI pilot study involving the NAO robot, this research demonstrates how facial expression analysis and speech emotion recognition can be employed to detect and address negative emotions in real time, fostering positive emotional engagement. The emotion recognition system combines handcrafted and deep learning features for facial expressions, achieving an 85% classification accuracy during real-time CRI, while speech emotions are analyzed using acoustic features processed through machine learning models with an 83% accuracy rate. Offline evaluation of the combined emotion dataset using a Dynamic Bayesian Mixture Model (DBMM) achieved a 92% accuracy for facial expressions, and the multilingual speech dataset yielded 98% accuracy for speech emotions using the DBMM ensemble. Observations from psychological and technological aspects, coupled with statistical analysis, reveal the robot’s ability to transition negative emotions into neutral or positive states in most cases, contributing to emotional regulation in children. This work underscores the potential of emotion-aware robots to support therapeutic and educational interventions, particularly for pediatric populations, while setting a foundation for developing personalized and empathetic human–machine interactions. These findings demonstrate the transformative role of affective computing in bridging the gap between technological functionality and emotional intelligence across diverse domains.

## 1. Introduction

Human behavior and cognition emerge from a complex interplay between perception, cognition, emotions, and actions. Affective computing, which leverages interdisciplinary insights from neuroscience, psychology, and artificial intelligence, explores these connections to create systems that can recognize, interpret, and respond to human emotions in real time. This field is transforming human–technology interaction by integrating emotional awareness into adaptive AI systems, allowing technology to respond not only to cognitive but also to emotional states, leading to more personalized and supportive user experiences.

The interdisciplinary nature of affective computing fosters collaboration across fields such as neuroscience, psychology, computer science, and engineering, driving advancements that enable technologies to cater to individual emotional responses, preferences, and needs. By enabling systems to tailor content, user interfaces, and interactions, affective computing has the potential to optimize user engagement, satisfaction, and outcomes in diverse settings. Unlike traditional approaches to human–computer interaction, which focus on general usability and efficiency, affective computing emphasizes the dynamic interaction between perception, emotion, and cognition to create emotionally intelligent systems that adapt based on real-time feedback.

Affective computing encompasses key components that drive this field’s innovation:

- Emotion–Cognition Integration: Recognizing that perception, emotion, and cognition shape each other in complex ways, affective computing systems seek to understand and leverage these interconnections to enhance interaction quality and user experience [1,2].
- Adaptive Systems: Emotion-aware technologies are designed to detect and respond to changes in users’ emotional states and adapt accordingly, providing personalized feedback and interventions that cater to users’ unique needs [3,4].
- Feedback and Intervention: By offering users real-time feedback based on their emotional states, affective computing technologies promote positive emotional experiences and enhance decision-making, especially in challenging or stressful scenarios [5,6].
- Human–Technology Interaction: Affective computing improves human–computer interaction by adjusting system responses based on user feedback, creating seamless, empathetic, and effective interactions that promote emotional regulation and engagement [7,8].

Recent advancements in machine learning, including artificial neural networks, deep learning, and biologically inspired algorithms, have greatly expanded our ability to detect and analyze emotional patterns [9,10]. Using multimodal data such as speech, facial expressions, text sentiment, and even neuroimaging data like EEG, affective computing systems can accurately recognize emotions and identify nuanced transitions in emotional states [11,12]. This multimodal approach enriches our understanding of human experience and enables highly accurate emotion recognition in adaptive AI systems.

The applications of affective computing are far-reaching, with significant potential to transform fields such as healthcare, business, and human–robot interaction. In healthcare, affective computing can support early mental health interventions by identifying emotional distress and providing tailored support. In business, it enables customer service systems to gauge customer satisfaction through analysis of vocal, textual, and facial cues, thus improving customer interactions and feedback systems. In human–robot interaction, affective computing facilitates empathetic interactions in applications such as elder care and social development interventions for children with autism spectrum disorder (ASD).

Affective computing has become integral to our daily interactions, enhancing virtual assistants, smart homes, and clinical interventions by incorporating emotional awareness. As AI and machine learning increasingly model principles of human behavior and emotion, we are building a future where technology is seamlessly integrated with human experience, enriching lives in unprecedented ways.

As affective computing advances, it holds transformative potential for many domains. The integration of affective computing into large language models (LLMs), such as OpenAI’s ChatGPT, represents a new era of human–machine interaction [13,14].

Key areas of impact include the following:

- Personalized Learning and Assistance: By recognizing and responding to users’ emotional cues, LLMs can provide tailored content and recommendations, fostering engaging and effective educational experiences [15].
- Enhanced Human–Computer Interaction: Emotion-aware language models enable empathetic and contextually aware interactions, where systems not only understand but adapt to users’ emotions, creating more meaningful engagements [16].
- Mental Health Support: Affective computing offers promise in developing AI-driven mental health support systems, capable of detecting early signs of distress and providing customized coping strategies or connecting users with resources [17].
- Empathetic Customer Service: Businesses can leverage affective computing for personalized, empathetic customer service, where chatbots understand and respond to emotions, driving higher customer satisfaction [18].
- Creative Expression and Innovation: By enabling emotionally aware interactions, affective computing empowers artists, writers, and creators with tools for new forms of expression and innovation [19].

Affective computing has become a key component in child–robot interaction (CRI), enabling social robots to recognize, interpret, and respond to children’s emotional and cognitive states. By integrating affective computing, robots can foster engagement, personalize learning experiences, and provide emotional support, making human–robot interaction more natural and meaningful. This section reviews key research on affective computing in CRI, engagement detection, and long-term interaction. The authors in [20] provide a comprehensive review of social robots in education, emphasizing how affective computing enhances robot-assisted learning by recognizing student emotions and adapting interactions accordingly. Their study highlights the importance of emotion-aware robots in improving motivation and learning outcomes, while also addressing challenges such as personalization and ethical considerations. Engagement is a crucial factor in affective child–robot interaction, as emotionally responsive robots foster more immersive and effective learning experiences. Castellano et al. [21] propose a framework to detect user engagement based on characteristics of tasks and social interactions. Their findings demonstrate that recognizing affective cues, such as facial expressions and social behavior, enables robots to adapt their responses dynamically, improving user interaction and engagement. Affective adaptation in long-term CRI remains a challenge. The authors of [22] discuss the need for robots to maintain children’s interest over time, emphasizing that robots must adapt to individual emotional patterns and learning preferences. Their study suggests that robots capable of emotional learning and contextual adaptation foster stronger and more meaningful relationships with children. Leite et al. [23] further explores long-term interactions with empathic social robots, focusing on how robots can develop emotional intelligence to sustain engagement. The study highlights that robots capable of recognizing and responding to users’ emotional states contribute to more effective learning and social interactions, ultimately enhancing the user experience. Furthermore, Wang et al. [24] examine the effectiveness of educational robots in improving learning outcomes, stressing the role of affective computing in tailoring instruction to student emotional and cognitive needs. Their findings confirm that emotion-aware robots improve motivation and the retention of learning. However, the study also points out the challenges of integrating affective computing into traditional educational frameworks and ensuring real-time emotional adaptation. In general, affective computing plays a crucial role in CRI, enabling robots to establish deeper connections with children by recognizing and responding to their emotions. Although significant progress has been made in engagement detection, emotion-aware learning, and long-term adaptation, future research must focus on refining affective models, improving real-time emotional recognition, and addressing ethical considerations to maximize the benefits of child–robot interaction. Therefore, affective computing is poised to reshape our interactions with machines, creating empathetic and adaptive systems that promote well-being and creativity. By enabling AI to understand and respond to human emotions, we can build a future where technology not only serves functional needs but also enriches human experience in an interconnected world.

Building upon these foundational concepts, this study focuses on applying affective computing principles to child–robot interactions in pediatric settings. By leveraging emotion recognition technologies, we aim to design adaptive robotic behaviors capable of responding to children’s emotional states in real time. Using the NAO robot, we explore how facial expression analysis and speech emotion detection can be integrated to facilitate dynamic emotional engagement, fostering positive interactions and supporting emotional regulation. Through a combination of advanced machine learning techniques and clinical observations, we investigate how emotion-aware robots can enhance user experiences and address unique emotional needs. This research underscores the potential of affective computing to transform human–machine interaction by creating empathetic systems tailored to individual preferences and emotional contexts. Thus, the contributions of this paper can be listed as follows:

- Emotion-aware CRI framework: A novel integration of emotion-aware technology in child–robot interactions, demonstrating the effectiveness of affective computing models in the real-time classification of facial expressions, speech emotions, and text sentiment.
- Multisensorial Data Analysis: Data analysis and insights into the psychological and behavioral impact of such systems, highlighting their potential for fostering emotional engagement, regulating emotions, and delivering societal benefits in clinical and educational contexts.

The remainder of this paper is organized as follows: Section 2 explores the foundations of emotion, tracing its biological origins and linking them to advancements in emotion-aware technology. Section 3 outlines the experimental setup, detailing the design of child–robot interaction sessions and the computational methods employed for analyzing facial expressions, speech emotions, and text sentiment. Section 4 presents the results, accompanied by an in-depth discussion of the findings, highlighting both technological performance and psychological insights. Finally, Section 5 concludes the study by summarizing the key contributions and proposing future directions to advance the integration of emotion-aware technology in real-world applications.

## 2. Foundations of Emotion: From Biological Roots to Technological Frontiers

### 2.1. Neuroscience of Emotion

Emotions are complex psychological and physiological phenomena that involve the interaction of various brain regions, neurotransmitters, and hormonal systems. The limbic system, which includes structures like the amygdala, hippocampus, and hypothalamus, plays a crucial role in processing emotions, memory formation, and regulating physiological responses to stress. Understanding the neurobiological underpinnings of emotions can provide valuable insights into how they influence cognitive processes, behavior, and decision-making [25,26]. Emotions significantly impact decision-making processes by influencing attention, memory, judgment, and risk perception. Emotional states can bias individuals towards certain choices, leading to decisions that may not always align with rational or logical reasoning. For example, positive emotions can enhance creativity and openness to new ideas, while negative emotions may narrow focus and increase risk aversion. By understanding how emotions shape decision-making, we can develop strategies to mitigate biases and make more informed choices [27,28]. Emotions play a crucial role in shaping behavior and social interactions, influencing how we perceive and respond to others, communicate needs, and navigate social dynamics. For instance, emotions like empathy, compassion, and gratitude foster prosocial behavior and cooperation, while anger, fear, or jealousy may lead to aggression, avoidance, or withdrawal. By understanding the interplay between emotions and behavior, we can promote positive social interactions, conflict resolution, and emotional well-being in individuals and communities [29,30,31,32,33].

Emotion regulation refers to the ability to monitor, evaluate, and modify one’s emotional reactions in response to internal and external stimuli. Effective emotion regulation skills are essential for mental health, interpersonal relationships, and overall well-being [34,35]. Affective computing leverages insights from neuroscience, psychology, and technology to develop interventions and tools that facilitate emotion regulation. By enhancing individuals’ capacity to regulate their emotions, we can improve resilience, adaptive coping strategies, and psychological flexibility. Emotions can sometimes bypass reasoning, directly influencing actions, particularly in situations requiring rapid responses for survival or adaptation. This comprehensive representation captures the complex and dynamic interaction between perception, emotions, cognition, and behavior. Bayesian reasoning using prior knowledge can mirror brain processes, merging senses to enhance perception, facilitating the recognition of emotions, which subsequently influence decision-making and behavioral responses in interactions with the environment [36,37,38,39,40].

Affective computing offers a multidisciplinary approach to understanding and harnessing the power of emotions in human cognition, behavior, and decision-making. By integrating insights from neuroscience, psychology, and technology, we can develop innovative solutions to enhance emotion regulation, promote mental health, and foster positive social relationships in today’s increasingly complex and interconnected world [41].

### 2.2. Common AI/Machine Learning Techniques for Affective Computing

Advancements in affective computing leverage a diverse array of AI and machine learning (ML) techniques, spanning both deep learning and classical methodologies. Deep learning techniques, particularly Convolutional Neural Networks (CNNs) and Recurrent Neural Networks (RNNs), are prominent in this domain due to their ability to simulate human-like learning processes. In image classification tasks, CNNs excel by extracting hierarchical patterns and features through multiple layers, enabling them to discern intricate details and nuances within images. Similarly, in audio analysis for speech emotion recognition [42], features extracted from time–frequency spectrograms provide rich representations of audio signals, which are then processed by deep neural networks to capture subtle emotional cues and patterns [43].

In contrast, classical machine learning techniques are also widely employed, particularly in tasks such as sentiment analysis. Pretrained models, such as Bidirectional Encoder Representations from Transformers (BERT), offer powerful tools for natural language processing, allowing for efficient sentiment analysis and emotion detection in textual data [44]. EEG data analysis using AI also plays a crucial role in understanding brain activity and emotion regulation [45]. Signal processing techniques, including wavelet transforms, extract frequency-based features from EEG signals, facilitating the identification of neural correlates of emotions. These features, along with statistical measures derived from frequency bands, are then utilized by various classifiers, ranging from CNNs to classical machine learning algorithms like Support Vector Machines (SVMs) and Random Forests (RFs), to classify emotional states.

Perception is inherently multisensory [46], where the integration of information across multiple sensory modalities enhances the robustness of estimations and solves ambiguities. If input from a single modality is insufficient for reliable assessment, the brain seamlessly integrates data from various modalities to form a more accurate perceptual representation. Perception, as a multisensory process, extends its relevance to emotion recognition by incorporating cues from various sensory modalities [47].

Measuring uncertainties from multiple modalities and data fusion are essential components in multimodal approaches [48]. Techniques such as Bayesian inference [49] and ensemble learning are employed to combine information from multiple sensory modalities, enhancing the robustness and accuracy of emotion recognition systems. Additionally, AI models, including pretrained architectures and novel deep learning frameworks, continue to evolve, driving advancements in research and real-world applications of affective computing. Data fusion techniques are essential, particularly in the domain of multisensory integration [50]. Early fusion [51], also known as feature-level fusion, combines raw data from multiple sources into a single representation at the earliest stage of processing, preserving the integrity of the original data but with potential computational load and complexity. On the other hand, late fusion [52], or decision-level fusion, processes data from individual sources separately and combines results later, allowing flexibility in handling heterogeneous data sources and accommodating varying levels of reliability and uncertainty. This approach enables nuanced interpretations of emotional cues and enhances system robustness.

### 2.3. Emotion Regulation and Technology

Technology can play a significant role in emotion regulation by providing tools and interventions that help individuals better understand, manage, and express their emotions. For instance:

- Emotion Tracking Apps: Mobile applications equipped with sensors can track physiological indicators like heart rate, skin conductance, and facial expressions to provide real-time feedback on emotional states [53]. This information helps users identify triggers, recognize patterns, and implement coping strategies to regulate their emotions effectively.
- Virtual Reality (VR) Therapy: VR technology offers immersive environments where individuals can engage in guided relaxation exercises, exposure therapy, or mindfulness meditation to alleviate stress, anxiety, or phobias [54].
- Biofeedback Devices: Wearable biofeedback devices, such as smartwatches or EEG headbands, monitor physiological signals associated with stress and relaxation [55].
- Chatbots and Virtual Assistants: AI-powered chatbots and virtual assistants equipped with natural language processing capabilities can offer empathetic responses and support individuals in managing their emotions [56].
- Biometric Feedback Systems: These systems enable users to visualize emotional states through interactive displays based on physiological data [57].

Technology has the potential to transform emotion regulation by providing innovative tools, interventions, and support systems that enhance self-awareness, coping skills, and mental well-being. By harnessing the power of technology, individuals can navigate their emotions more effectively, leading to improved mental health outcomes and a greater sense of emotional balance and resilience.

### 2.4. Ethical Considerations with Technological Applications in Affective Computing

As technology continues to advance, particularly in affective computing, addressing the ethical implications of these innovations becomes imperative. Emotion recognition technologies, which utilize audio, video, text, brain activities, and attentional indicators, raise concerns regarding privacy, consent, fairness, and potential biases. Key ethical considerations include the following:

- Privacy and Consent [58,59,60]: Ethical concerns revolve around the privacy and informed consent of individuals whose emotions are being monitored and analyzed [61]. The collection and processing of sensitive emotional data require transparent policies and robust safeguards to ensure user autonomy, confidentiality, and data security.
- Fairness and Bias: There is a risk of algorithmic bias and unfair discrimination in emotion recognition systems [62]. Biases in training data or model algorithms can lead to inaccuracies and disparities, disproportionately affecting certain demographic groups.
- Accountability and Responsibility: With the increasing integration of affective computing in various domains, accountability among developers, practitioners, and policymakers is essential [63].

Impact on healthcare, education, business, and marketing highlights the transformative potential of affective computing. In healthcare, these technologies facilitate early mental health interventions, improve patient engagement, and enhance empathetic care. In education, emotion-aware systems support personalized learning experiences. In business, emotion analytics inform product design and customer experience strategies. Ensuring ethical development and deployment is crucial for inclusive, equitable, and empathetic advancements in affective computing.

### 2.5. Advancing Beyond the State of the Art

This work advances beyond the current state of the art by integrating multimodal emotion recognition—encompassing facial expressions, speech emotions, and text sentiment—within real-time child–robot interactions. Unlike previous studies, this approach combines robust computational models with psychological insights to provide a comprehensive understanding of children’s emotional and behavioral responses during structured activities. The findings highlight the system’s capability to elicit and sustain positive emotional engagement while minimizing negative responses, demonstrating its potential for therapeutic and educational applications. This interdisciplinary perspective bridges technological innovation and psychological analysis, offering a practical framework for emotion-aware systems with real-world societal impact.

## 3. Real-World Case Study and Methods

This study aimed to explore CRI by using the NAO robot as a facilitator of emotional engagement in pediatric settings. The goal was to assess emotional responses during structured interactions and to develop adaptive robotic behaviors based on detected emotions.

### 3.1. Experimental Design

The NAO robot engaged 14 children (aged 5–8 years) in a 10 min scripted interaction. The session included five stages, as presented in Table 1.

**Figure 1:**
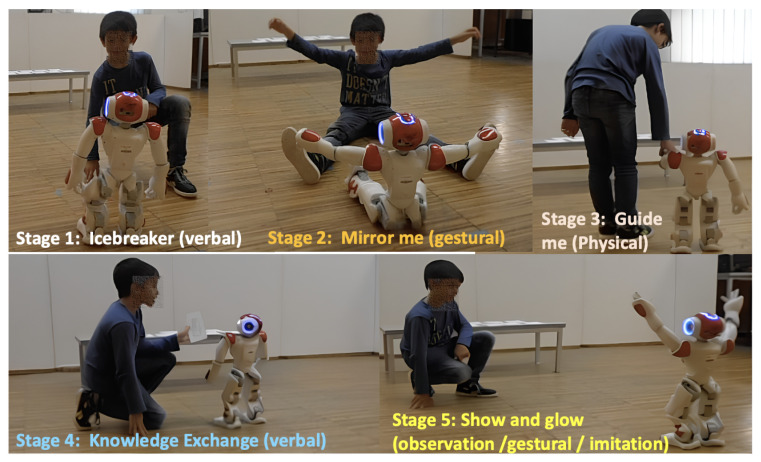
Overview of a child–robot interaction session with specific frames from each stage defined.

The type of session described in Table 1 was designed with the assistance of clinical psychologists to detect emotional expressions during child–robot interactions across various tasks that require social interaction and sustained attention. Figure 1 shows the child–robot interaction session and its stages.

Two technical modules operated in parallel during the sessions:

- Facial Expression Analysis: The robot analyzed facial expressions using an early fusion of handcrafted features engineering with deep learning features (VGG16) with two classical machine learning algorithms, Support Vector Machine (SVM) and Random Forest (RF), fused via soft late fusion using a probabilistic ensemble model. When a negative expression (e.g., sad, disgusted, or angry) persisted in the past minute as predominant, the robot paused its activity and initiated a dialogue followed by actions such as playing a song or dancing, to address the child’s emotional state.
- Speech Emotion Detection: Speech emotions were detected during dialogues using acoustic feature analysis and machine learning classifiers, an ensemble consisting of a Convolutional Neural Network (1D-CNN) and a Multilayer Perceptron network (MLP). When a negative emotion was identified as predominant over other emotions in a period of time, the robot paused its activity and reacted (by playing a song or dancing) to mitigate the negativity.

After addressing the detected negative emotion, the robot resumed the stages of the session. These integrated approaches to facial and speech emotion recognition illustrate the power of affective computing in creating systems capable of accurately interpreting human emotions.

The defined Experimental Hypotheses and Factorial Design for our CRI sessions are presented below. To systematically evaluate the interaction between the robot and the child, we formulated the following hypotheses:

- **H1**: The robot’s adaptive emotional responses will lead to increased engagement and positive emotions during structured interaction phases.
- **H2**: Differences in engagement levels will be observed based on gender and parental involvement.
- **H3**: The accuracy of emotion recognition will remain stable across different phases, validating the robustness of the multimodal system.

The study follows a 2 × 3 factorial design, with the following variables:

- Independent variables: (1) Gender (Male/Female) and (2) Interaction phase (e.g., Icebreaker, Guided Steps, Show and Glow).
- Dependent variables: (1) Emotional valence (positive/negative), (2) Engagement score (measured via observation and system logs), and (3) System recognition accuracy.

This structured approach ensures clarity in statistical analysis and enhances result interpretation as detailed in the Results section.

Using machine learning models and fusion techniques, affective computing provides critical tools for improving human–machine interaction and providing emotionally intelligent personalization in diverse applications.

### 3.2. Ethical Considerations for the Experiments

This study was approved by the University’s Ethics Committee and adheres to ethical guidelines for child studies involving AI-based interventions by using consent forms and taking into consideration data privacy, as explained in Section 2.4. To mitigate potential negative emotional responses, the system included real-time monitoring, allowing the facilitator to intervene if distress signals were detected. Additionally, parents remained present during the session, ensuring emotional security for the children. Age appropriateness was also considered: participants were aged 5 to 8 years, a range established in prior CRI studies [64,65]. Younger children (below 5) were excluded due to challenges in interacting with humanoid robots at a cognitive and social level.

### 3.3. Psychological Observations and Data Analysis

Together with a clinical psychologist, we monitored the sessions and assessed children’s emotional responses, engagement, and acceptance. The Emotional Assessment Scale (EAS) was used to quantify emotions, and parental questionnaires provided additional insights into child–parent emotional concordance.

Emotional responses were measured using intensity scales (0–100). The following statistical techniques were applied:

- Z-Scores: Z-scores are calculated to measure how far a data point (e.g., emotional intensity) deviates from the mean, expressed in units of standard deviation. Z-scores helped to identify significant outliers in emotional responses and compare differences across groups (e.g., children vs. mothers).
- *p*-Values: The *p*-value represents the probability that the observed difference between groups occurred by chance. A *p*-value below 0.05 was considered statistically significant, indicating that differences in emotional responses between children and mothers were unlikely to be due to random variation.
- Wilcoxon Signed-Rank Test: This nonparametric test was used to compare paired emotional intensity scores between children and their mothers. It is suitable for small sample sizes and does not assume a normal distribution of data.
- Cronbach’s Alpha: Internal consistency was assessed to ensure the reliability of emotional intensity measurements. Values above 0.7 indicated acceptable reliability, with α=0.87 for children and α=0.68 for mothers.

To ensure ethical compliance in conducting this pilot project, we adhered to established ethical research protocols. Before initiating the child–robot interaction experiments, we obtained approval from the institutional ethics review board. The parents of all participating children were provided with detailed information about the study, including its objectives, procedures, potential benefits, and measures to protect participant confidentiality. Informed consent forms were distributed to the parents or legal guardians, who signed them to authorize their children’s participation. These forms explicitly permitted the use of the collected data for research purposes while ensuring anonymity and secure data handling. This rigorous ethical approach ensured the well-being of the participants and the integrity of the research process.

### 3.4. Affective Computing: Facial and Speech Emotion

Affective computing, bridges the gap between emotional intelligence and technology by leveraging advanced algorithms to detect, interpret, and respond to human emotions in real time. Emotion recognition, as discussed in earlier sections, is a cornerstone of human–machine interaction, as emotions play a critical role in shaping communication, decision-making, and engagement. In human interactions, facial expressions and speech serve as primary channels for conveying emotional states, making them highly relevant modalities for emotion recognition in affective computing systems. Facial expressions provide visual cues that reflect both conscious and subconscious emotional responses, while speech conveys affective information through tone, pitch, and rhythm, offering insights into a speaker’s emotional state. Together, these modalities enable a deeper understanding of human emotions, fostering more intuitive and empathetic interactions between users and machines.

Facial expression analysis plays a pivotal role in emotion recognition by enabling systems to understand and respond to user emotions more effectively. A hybrid approach was adopted [66,67,68,69], integrating early fusion at the feature level. This methodology combines handcrafted geometric features derived from facial landmarks, such as distances between landmarks, angles, and log-covariance of pairwise landmark distances, with features extracted from a Histogram of Gradients (HoG), Local Binary Patterns (LBPs), and deep learning features derived from the VGG16 model. To enhance feature selection, the information gain criterion was employed, isolating the most relevant features for training classifiers. Two classification algorithms were utilized: a linear SVM and an RF model. The outputs of these classifiers were fused using an ensemble approach, specifically a Dynamic Bayesian Mixture Model (DBMM) [70], which combines the strengths of the individual classifiers to improve accuracy [71].

Speech emotion recognition, another critical aspect of affective computing, involves analyzing audio signals to identify emotional cues. This process begins with feature extraction, encompassing 48 Mel-frequency Cepstral Coefficients (MFCCs), 128 mel spectrogram features, 12 chroma features, 6 tonnetz features, and additional metrics such as pitch, energy, zero-crossing rate, and RMS energy. Statistical measures—including mean, standard deviation, minimum, maximum, and median—are computed for these features, resulting in a total of 950 features per audio sample. Two classification models were developed for speech emotion recognition: an MLP and a 1D-CNN. The MLP comprises three fully connected layers, with 320 neurons in the first layer, 192 neurons in the second layer, and a final softmax layer with 4 output neurons corresponding to emotion classes. This architecture contains 95,303 trainable parameters. The 1D-CNN employs a 1D convolutional layer with 128 filters, followed by max pooling, flattening, and a dense layer with 4 output neurons, totaling 22,663 trainable parameters. Both models were trained using categorical cross-entropy loss and optimized with the Adam optimizer. To further improve classification accuracy, the outputs of the MLP and 1D-CNN were fused using the DBMM approach [70], effectively integrating the strengths of both models via an weighting approach based on entropy to measure the classifier with higher confidence. Transfer learning and calibration, including a brief recording session for user-specific adjustments, enhanced the framework’s performance for classifying emotions from short audio clips of approximately 7 s.

Algorithms 1 and 2 present the facial expression and speech emotion classification frameworks.
**Algorithm** **1** Facial Expression Recognition Using DBMM Ensemble1:**Input:** Face image of size 224×2242:**Output:** Predicted facial expression class3:**Step 1: Preprocessing the Image**4:     Detect facial landmarks using a pretrained landmark detector (DLib or MediaPipe)5:     Normalize the face image to ensure consistent scale and orientation.6:     Resize the image to 224×224 (input size for VGG16).7:**Step 2: Feature Extraction**8:    *Handcrafted Geometric Features:*9:    Extract distances among facial landmarks, angles from landmark triangles, and log-cov matrices for landmarks pairwise distances.10:     Compute HoG features over the face image.11:     Compute LBP features from patches of the face image.12:     *Deep Learning Features (VGG16):*13:     Pass the preprocessed image through the VGG16 model (pretrained) and extract features from its fully connected layers.14:**Step 3: Feature Fusion**15:     Concatenate all extracted features into a single feature vector:16:    $F=\mathrm{Concat}(F_{\mathrm{geo}},F_{\mathrm{HoG}},F_{\mathrm{LBP}},F_{\mathrm{VGG}16})$17:    where:18:    $F_{\mathrm{geo}}$ are the geometric features,19:    $F_{\mathrm{HoG}}$ are the HoG features,20:    $F_{\mathrm{LBP}}$ are the LBP features,21:    $F_{\mathrm{VGG}16}$ are the VGG16 deep learning features.22:**Step 4: Feature Selection**23:    Apply Information Gain to select the most relevant features:24:    $IG\left(F_{i}\right)=H\left(C\right)-H\left(C|F_{i}\right)$25:    where:26:    $IG(F_{i})$ is the information gain of feature $F_{i}$,27:    H(C) is the entropy of class labels,28:    $H(C|F_{i})$ is the conditional entropy given feature $F_{i}$.29:**Step 5: Train SVM and RF Classifiers**30:    *Train Support Vector Machine (SVM):*31:    $\min_{w,b}\frac{1}{2}{∥w∥}^{2}$    subject to    $y_{i}\left(w\cdot x_{i}+b\right)\geq 1\;\;\;\;\forall i$32:     *Train Random Forest (RF):*33:    ${\hat{y}}_{\mathrm{RF}}=\mathrm{Majority}\;\mathrm{Vote}\;\mathrm{from}\;100\;\mathrm{Decision}\;\mathrm{Trees}$34:**Step 6: Individual Predictions by SVM and RF**35:    Predict with SVM:36:    ${\hat{y}}_{\mathrm{SVM}}=\mathrm{SVM}\left(F_{\mathrm{selected}}\right)$37:    Predict with RF:38:    ${\hat{y}}_{\mathrm{RF}}=\mathrm{RF}\left(F_{\mathrm{selected}}\right)$39:**Step 7: Ensemble Prediction with DBMM**40:    Compute Posterior Distribution using DBMM:41:    $P\left(C^{t}|C^{t-1:t-T},A^{t}\right)=\frac{\prod_{k=t}^{t-T}P\left(C^{k}|C^{k-1}\right)\times \sum_{i=1}^{2}w_{i}^{t}\times P_{i}\left(A^{t}|C^{t}\right)}{\sum_{j=1}^{\mathrm{classes}}\left[\prod_{k=t}^{t-T}P_{i,j}\left(C^{k}|C^{k-1}\right)\times \sum_{i=1}^{2}w_{i}^{t}\times P_{i,j}\left(A^{t}|C^{t}\right)\right]}$42:    where:43:    $P(C^{k}|C^{k-1})$ is the prior from previous posteriors,44:    $w_{i}^{t}$ is the weight for each classifier (SVM and RF),45:    $P_{i}\left(A^{t}|C^{t}\right)$ is the likelihood from each classifier at time *t*.46:    Compute Weights for Classifiers using Inverse Entropy:47:    $w_{i}=1-\left(\frac{-\sum_{k=1}^{s}P_{i,k}\left(\cdot \right)\log\left(P_{i,k}\left(\cdot \right)\right)}{\sum_{i=1}^{2}\left(-\sum_{k=1}^{s}P_{i,k}\left(\cdot \right)\log\left(P_{i,k}\left(\cdot \right)\right)\right)}\right)$48:    where:49:    $P_{i,k}$ is the class conditional probability of the *i*-th classifier,50:    *s* is the number of posteriors considered.51:**return:** Predicted facial expression class
**Algorithm** **2** Speech Emotion Recognition Using MLP, 1D-CNN, and DBMM Ensemble1:**Input:** Audio signal of emotional speech2:**Output:** Final predicted emotion class: {angry, happy, surprise, neutral}3:**Step 1: Feature Extraction**4:Extract features from the audio signal:5:     MFCCs: 48 features, $C_{n}=\sum_{m=0}^{M-1}M_{m}\left(k\right)\cdot \cos\left(\frac{\pi }{M}\left(m+\frac{1}{2}\right)n\right)$6:     Mel Spectrogram: 128 features, $\left(f,t\right)=\sum_{n=0}^{N}{\left|X\left(f,t\right)\right|}^{2}$7:     Chroma STFT: 12 features, $C_{i}\left(t\right)=\sum_{k=0}^{K}P\left(k,t\right)\cdot \delta \left(f_{k}-f_{i}\right)$8:    Tonnetz: 6 features9:    Additional Features: Pitch, Energy, Zero-Crossing Rate, RMS Energy (4 features)10:     Compute statistical features (mean, std, min, max, median) for MFCCs, Mel Spectrogram, and Chroma, Total features = 950.11:     Normalize the extracted features.12:**Step 2: MLP Model Training and Prediction**13:     *MLP Architecture:*14:     Input Layer: Flattened input of 950 features.15:     Dense Layer 1: 320 neurons, ReLU activation.16:     Dense Layer 2: 192 neurons, ReLU activation.17:     Output Layer: Softmax layer with 4 neurons (emotion classes).18:     The MLP computes the predicted emotion class:19:     ${\hat{y}}_{\mathrm{MLP}}=\arg\;\max\left(\mathrm{softmax}\left(W_{2}\cdot \mathrm{ReLU}\left(W_{1}\cdot x+b_{1}\right)+b_{2}\right)\right)$20:     where x is the feature vector, $W_{1},W_{2}$ are the weights, and $b_{1},b_{2}$ the biases.21:**Step 3: 1D-CNN Model Training and Prediction**22:     Input: Reshaped input features of size (950, 1)23:     *1D-CNN Architecture:*24:     Conv1D Layer: 128 filters, kernel size 3, ReLU activation.25:     MaxPooling1D Layer: Pool size 2.26:     Flatten Layer.27:     Output Layer: Softmax layer with 4 neurons (emotion classes).28:     The 1D-CNN computes the predicted emotion class:29:     ${\hat{y}}_{\mathrm{CNN}}=\arg\;\max\left(\mathrm{softmax}\left(W_{\mathrm{CNN}}\cdot \mathrm{Flatten}\left(\mathrm{Conv}1D\left(x_{\mathrm{reshaped}}\right)\right)+b_{\mathrm{CNN}}\right)\right)$30:**Step 4: Ensemble Prediction using DBMM**31:     Combine the predictions (MLP and 1D-CNN) using DBMM.32:     The posterior probability for each class:33:     $P\left(C^{t}|C^{t-1:t-T},A^{t}\right)=\frac{\prod_{k=t}^{t-T}P\left(C^{k}|C^{k-1}\right)\times \sum_{i=1}^{2}w_{i}^{t}\times P_{i}\left(A^{t}|C^{t}\right)}{\sum_{j=1}^{\mathrm{classes}}\left[\prod_{k=t}^{t-T}P_{i,j}\left(C^{k}|C^{k-1}\right)\times \sum_{i=1}^{2}w_{i}^{t}\times P_{i,j}\left(A^{t}|C^{t}\right)\right]}$34:     where:35:     $P(C^{k}|C^{k-1})$ is the prior from previous posteriors,36:     $w_{i}^{t}$ is the weight for each classifier (MLP and CNN),37:     $P_{i}\left(A^{t}|C^{t}\right)$ is the likelihood from each classifier at time *t*.38:**Step 5: Final Prediction**39:     Predicted class: $\hat{y}=\arg\;\max P\left(C^{t}|C^{t-1:t-T},A^{t}\right)$40:**return:** Final predicted emotion class

### 3.5. Quantifying the Engagement Score

To assess the level of engagement during child–robot interaction sessions, an Engagement Score (*E*) was computed based on five key factors: proxemics, facial expressions, speech emotion, participation, and the need for caregiver presence. This score was designed to quantify observable behaviors and emotional states, providing a comprehensive measure of engagement.

#### 3.5.1. Factors and Their Measurements

- Proxemics *P*: This factor refers to the distance between the child and the robot during the interaction. Observers visually estimated the distance without technological aid, categorizing proximity into ranges (e.g., personal or social space). Distances were normalized to a 1–5 scale, where 1 represented the farthest distances (>3 m) and 5 the closest proximity (<0.5 m).
- Facial Expressions *F*: This factor reflects the emotional state of the child during interaction. The facial expression recognition algorithm was used to detect and classify emotions (happiness, neutrality, fear, etc.). The degree of emotional expressiveness and variability contributed to the score, which was normalized to a 1–5 scale, where 1 represented negative expressions, 3 neutral, and 5 highly expressive emotions (surprise/happiness).
- Speech Emotion *S*: Speech was analyzed using a speech emotion recognition algorithm, categorizing the children feedback provided to the robot after the performed activities (stages) into positive, neutral, and negative. The emotion and tone of the child’s speech sentiment were normalized to a 1–5 scale, where 1 represented predominantly negative tones and 5 predominantly positive tones.
- Participation *A*: This factor evaluates the child’s active involvement during the session. Observers recorded behaviors such as verbal interaction (e.g., speaking or responding to the robot), non-verbal interaction (e.g., gestures during activities and touching the robot), and task completion (e.g., successfully engaging in activities posed by the robot). Participation was scored from 1 (minimal or no interaction) to 5 (consistently active engagement).
- Mother’s Presence *M*: This factor reflects the independence of the child during the session. Observers noted whether the child required their mother or caregiver to remain present. Independence was normalized to a 1–5 scale, where 1 indicated constant caregiver presence and 5 full independence.

#### 3.5.2. Weights Assigned to Factors

Weights were empirically assigned to reflect the relative importance of each factor in assessing engagement. The final weights were as follows:

- $W_{P}=0.3$: Proxemics was weighted highest because physical proximity to the robot is a fundamental indicator of comfort and interaction willingness.
- $W_{F}=0.2$: Facial expressions were weighted second, as emotional expressiveness often correlates with engagement.
- $W_{S}=0.2$: Speech emotion received equal weight to facial expressions, reflecting the importance of verbal interaction.
- $W_{A}=0.2$: Participation was also weighted at 0.2, as active interaction behaviors are crucial for measuring engagement.
- $W_{M}=0.1$: Mother’s presence was weighted lowest, as it is considered a secondary factor indicative of independence rather than direct engagement.

The weights were determined through iterative observations and expert review, informed by previous research and pilot observations. This empirical approach ensured that weights reflected the observed contributions of each factor to overall engagement.

#### 3.5.3. Quantification of Engagement Score

The engagement score *E* was computed as a weighted sum of the normalized factor scores: $E=\sum_{i=1}^{5}W_{i}\cdot X_{i}$, where

- $X_{i}$: Normalized value of each factor (P,F,S,A,M).
- $W_{i}$: Weight assigned to each factor, such that: $\sum_{i=1}^{5}W_{i}=1$.

#### 3.5.4. Observer Training

Observers were trained to ensure the consistent scoring of proxemics and participation. Visual observations were guided by a structured rubric, and inter-observer reliability checks were conducted to minimize biases. This methodology provides a reproducible framework for quantifying engagement during child–robot interaction sessions, balancing the importance of behavioral and emotional factors in the overall engagement assessment.

## 4. Results

### 4.1. Technical Results: Data Analysis

#### 4.1.1. Evaluating Facial Emotion Recognition on Benchmark Datasets

To classify facial expressions, we used a dataset combining the KDEF dataset [72] and a Real Emotion dataset collected in our previous work [67]. The KDEF dataset (Karolinska Directed Emotional Faces) consists of 4900 images of 70 individuals (35 women and 35 men) displaying seven emotions: happiness, sadness, anger, fear, surprise, disgust, and neutral expressions. It is widely used for facial emotion recognition due to its controlled conditions and high-quality labeling.

The Real Emotion dataset [67] was built by asking participants to watch emotionally triggering video sequences, yielding a total of 30,600 frames. Participants consisted of six individuals (three males and three females, aged 22–38). The dataset contains RGB images annotated with ground truth labels based on expected reactions.

The merged dataset was used to train a DBMM ensemble model (combining SVM and RF classifiers), as explained in Section 3. The classification performance of the model on the training datasets is shown in Table 2.

When applied to real-time classification during child–robot interaction sessions, the model achieved an average classification accuracy of 85%, with over 90% accuracy for classifying happiness and neutral emotions. The images were primarily captured by the NAO robot’s camera. Additionally, auxiliary cameras in the environment were utilized to ensure continuous facial detection in cases where the robot’s camera was unable to capture the face due to head movements or occlusions during the interactions. This multi-camera setup enhanced the robustness of the facial expression analysis.

An important consideration is that while the primary dataset used for facial emotion recognition was trained on adult expressions, our model demonstrates strong generalization to children by leveraging low-level facial features, such as micro-movements of the mouth, eyebrows, and cheeks. These geometric attributes, combined with deep learning-based feature representations, enable the robust detection of emotional expressions, even in younger individuals. The use of the DBMM ensemble was also a key factor to improve classification accuracy. Furthermore, transfer learning presents a valuable approach for enhancing performance. As part of future work, we plan to fine-tune the model using a child-specific dataset collected during our experiments to further optimize accuracy and adaptability in recognizing children’s emotions.

**Figure 2:**
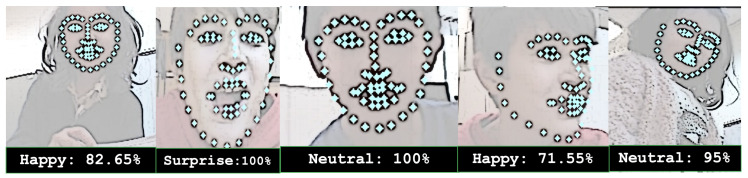
Examples of facial landmark detection and expression recognition performed on images captured by the NAO robot’s camera. The images are intentionally filtered to blur the children’s faces, ensuring their privacy.

Figure 2 presents sample frames captured by the NAO robot’s camera, showcasing the facial expression recognition algorithm in action. The algorithm detects facial landmarks and identifies expressions during CRI sessions.

#### 4.1.2. Evaluating Speech Emotion Recognition on Benchmark Datasets

The speech emotion model was trained using the EmoUERJ dataset [73] and the ESD (Emotional Speech Dataset) [74]:

- ESD: A multilingual emotional dataset containing over 35,000 recordings across five emotions: neutral, happy, sad, angry, and surprised.
- EmoUERJ: Brazilian Portuguese speech labeled with multiple emotional categories.

The DBMM model (combining 1D CNN and MLP) achieved superior performance compared to individual classifiers. The performance metrics on individual datasets are presented in Table 3. The performance metrics on the multilingual dataset (EmoUERJ + ESD) are shown in Table 4.

For real-time classification during child–robot interactions, the speech emotion model achieved an average accuracy of 83%. Audio clips ranging from 7 to 10 s were captured using the NAO robot’s built-in microphones, either in response to the robot’s questions or when the child initiated speech. This setup ensured reliable audio capture for effective emotion classification during the interactions.

#### 4.1.3. Data Analysis of Emotional Responses During the CRI Pilots

During the CRI sessions with 14 children, we analyzed facial expressions, speech emotions, and text sentiment feedback across the five stages of the session, as described in Table 1. After each stage, the robot prompted the children with a series of questions to gather their feedback on each concluded task. Examples of these questions included “Did you enjoy the last task?”, expecting a “yes”, “no”, or “more or less” response, and more open-ended questions like “What did you feel when performing the task?”, with emotion options such as joy, fear, boredom, sadness, anger, or normal. This would allow us to analyze their speech emotion and text sentiment by converting the audio to text and applying a sentiment analysis [70] of the provided sentence to identify positive, neutral, or negative sentiment given their answer, and also their facial expression when providing the answer, so that we could summarize their answer and the automated detection of their biophysical data. Table 5 presents the overall results detected by our affective computational models during the session. It presents the average values of all 14 children’s sessions.

The children’s responses were analyzed using multiple modalities. Speech emotions were detected directly from their vocal expressions, while their responses were converted from audio to text for sentiment analysis, leveraging the method described in [70]. This analysis classified their answers into positive, neutral, or negative sentiments. Simultaneously, we captured their facial expressions during the feedback process to complement the emotional analysis. By integrating these data streams, we summarized the children’s verbal and non-verbal emotional responses and the automated detection of their biophysical signals.

Table 5 presents the overall results detected by our affective computational models during the sessions, summarizing the average values across all 14 children. These results provide a comprehensive view of the children’s emotional and behavioral responses throughout the interaction.

**Figure 3:**
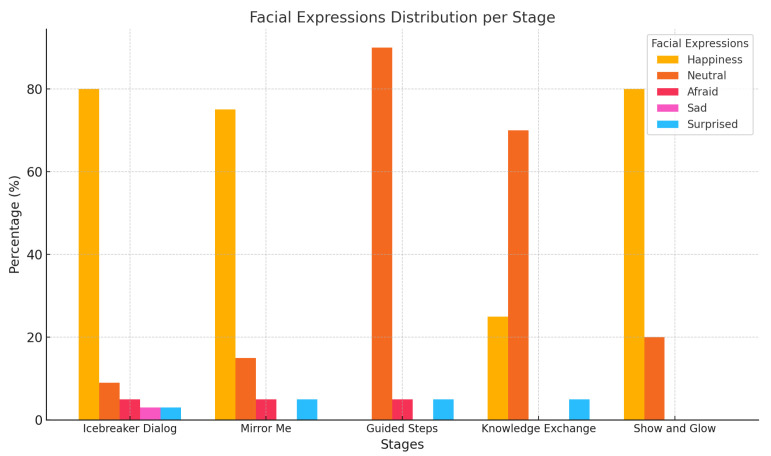
Facial expression distribution per stage. This figure illustrates the distribution of emotions, including happy, neutral, afraid, sad, and surprised, observed across the five stages of the child–robot interaction.

Figure 3 presents the distribution of facial expressions across the five stages of child–robot interaction. The emotions analyzed include happiness, neutral, fear, sadness, and surprise.

Key Observations from Facial Expression Analysis:

- Happy is most prevalent during the “Icebreaker Dialog”, “Mirror Me”, and “Show and Glow” stages, highlighting strong positive engagement in these interactive moments.
- Neutral expressions dominate the “Guided Steps” and “Knowledge Exchange” stages, indicating that these tasks required focus and attention rather than emotional arousal.
- Negative emotions, such as fear and sadness, are minimal across all stages, with minor peaks during the “Guided Steps” stage.

This distribution suggests that the child–robot interactions elicited primarily positive emotional responses, with neutral expressions reflecting concentration during more task-oriented stages.

**Figure 4:**
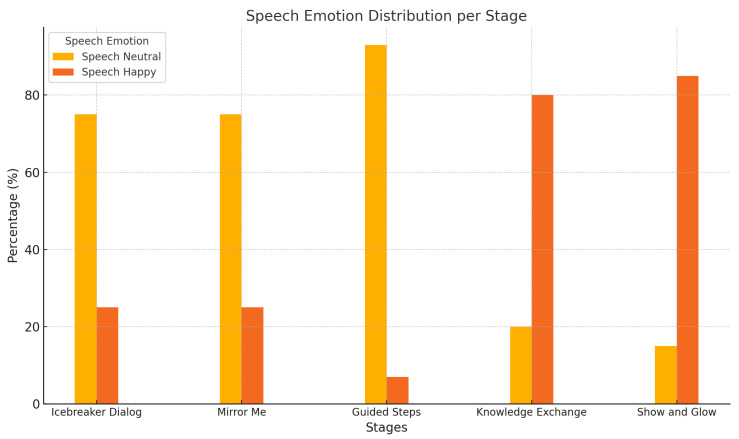
Speech emotion distribution per stage. Our approach could detect only neutral and positive emotions. Other emotions like fear, sadness, and anger were not detected.

Figure 4 focuses on the distribution of speech emotion, comprising neutral and happy categories, across the five stages of child–robot interaction.

Key Observations from Speech Emotion Analysis:

- Neutral speech dominates the earlier stages, such as “Icebreaker Dialog” and “Mirror Me”, reflecting a composed engagement as children interact with and respond to the robot.
- Happy speech significantly increases during the “Knowledge Exchange” and “Show and Glow” stages, showcasing heightened emotional involvement in the robot’s dynamic and entertaining activities.

The progression from neutral to happy speech across the stages emphasizes the robot’s ability to foster positive emotional engagement as interactions evolve.

**Figure 5:**
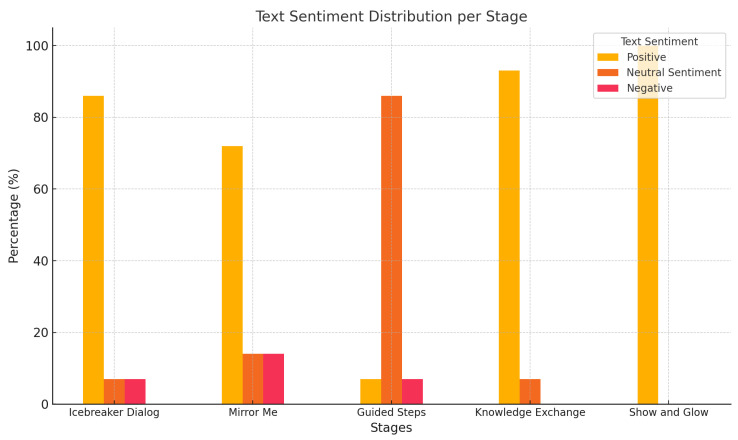
Text sentiment distribution per stage. Based on the sentiment analysis of text converted from speech, this figure shows the proportions of positive, neutral, and negative sentiments across the stages.

Figure 5 displays the distribution of text sentiment, based on feedback converted from speech to text and analyzed for positive, neutral, and negative sentiments.

Key Observations from Sentiment Analysis (Children’s Feedback):

- Positive sentiment overwhelmingly dominates across all stages, peaking at 100% in the “Show and Glow” stage, which involves an entertaining robot dance.
- Neutral sentiment is more prevalent in earlier stages, such as “Guided Steps”, where the structured nature of the activity prompted balanced emotional responses.
- Negative sentiment remains minimal throughout the sessions, indicating an overall favorable perception of the interaction.

These results highlight the effectiveness of the robot in eliciting positive emotional feedback and maintaining minimal negative sentiment during the activities. When the negative feedback is predominant, the robot just says: “I will play a song to cheer you up!” and plays a 30 s happy song as an attempt to improve the engagement and prepare the child for the next stage.

**Figure 6:**
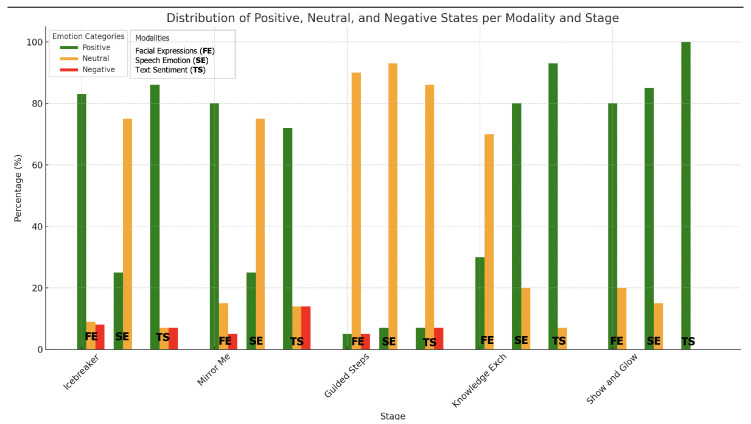
Summary of positive, neutral, and negative emotional states per stage. This figure aggregates the results from facial expressions, speech emotions, and text sentiment, summarizing the emotional states into positive (happy and surprised), neutral, and negative (sad, afraid, angry, and disgusted).

Figure 6 aggregates the results across facial expressions, speech emotion, and text sentiment, summarizing the emotional states into three categories: positive (comprising happy and surprised), neutral, and negative (comprising sad, afraid, angry, and disgusted).

Consequently, the following key observations were made:

- Positive emotions consistently dominate, peaking during the “Show and Glow” stage, where both facial expressions and text sentiment align to indicate high engagement and satisfaction.
- Neutral emotions are prominent in task-oriented stages, such as “Guided Steps” and “Knowledge Exchange”, reflecting the children’s focus and attention.
- Negative emotions are negligible across all stages, further affirming the robot’s ability to sustain positive interactions.

This summary emphasizes the effectiveness of child–robot interactions in fostering positive emotional states, maintaining engagement, and minimizing negative responses.

### 4.2. Psychological Observations Related to Mother-Child Emotions and Acceptance During CRI

The most frequent emotions in children were happiness (M=74.8; SD=10.3) and surprise (M=65.3;SD=17.4). Anxiety (M=29.2;SD=25.9) and fear (M=20.3; SD=19.1) were observed at higher levels in children than mothers, who reported lower means for these emotions. Table 6 summarizes the emotional responses.

**Figure 7:**
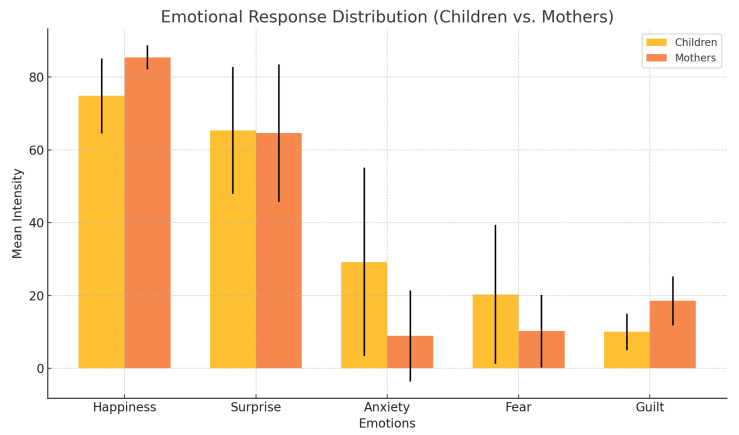
Emotional response distribution (children vs. mothers).

**Figure 8:**
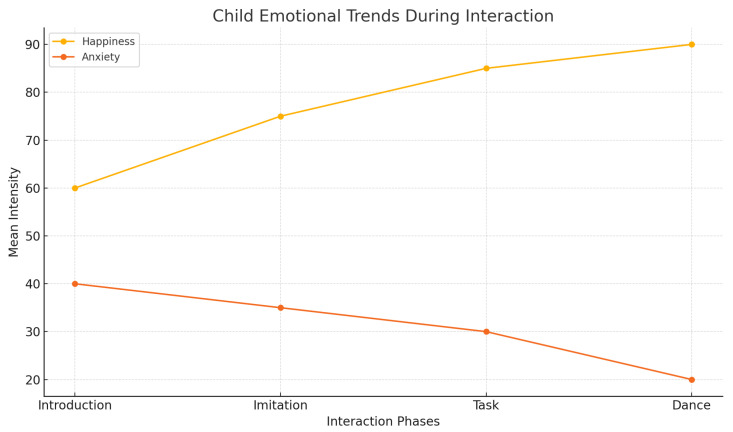
Child emotional trends during interaction.

**Figure 9:**
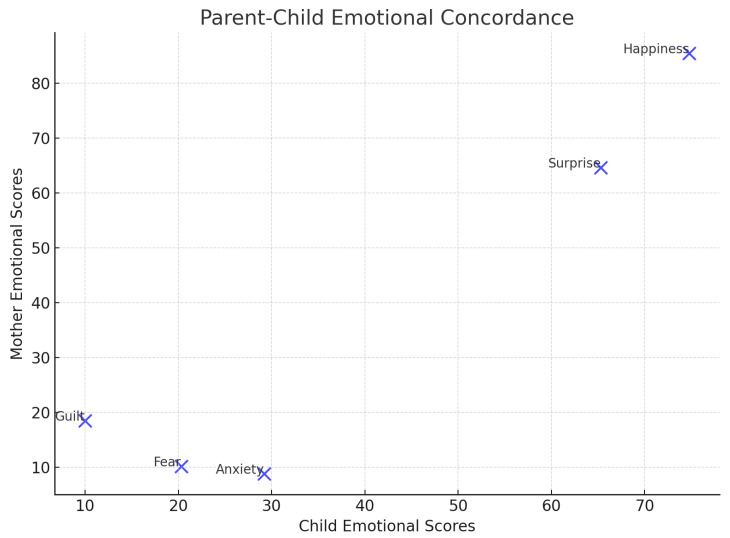
Parent–child emotional concordance.

Figure 7, Figure 8 and Figure 9 provide insights into emotional responses and concordance. They illustrate the mean intensity of emotional responses for both children and their mothers during the child–robot interaction sessions. We observed that happiness and surprise were the most frequently reported emotions, while anxiety and fear were more prominent in children than in mothers, with statistically significant differences as indicated by the *p*-values. Figure 8 shows the progression of children’s emotional states across different phases of the interaction (Introduction, Imitation, Task, and Dance). The graph demonstrates a steady increase in happiness and a concurrent decrease in anxiety, reflecting the effectiveness of the robot’s engagement strategies. Figure 9 presents a scatter plot comparing the emotional intensity scores of children and mothers, showing strong alignment for positive emotions such as happiness and surprise, but notable differences in negative emotions like anxiety and fear. These figures collectively emphasize the role of emotion-aware robot behaviors in influencing the emotional dynamics of child–robot interactions.

We observed the following situation during the CRI experiments:

- Three girls (aged 5, 5, and 6) experienced negative emotions such as fear, sadness, and anger, possibly due to their anxiety, for over a minute, given by the predominant emotion observed in the past minute. The robot successfully engaged them with dancing and music, transitioning their emotions to neutral or positive states.
- Some children displayed brief negative facial expressions lasting less than 5 s. These short-lived expressions did not trigger the robot’s intervention, as they were not the predominant emotional state observed within the past minute. The robot calculates the percentage duration of each detected emotion over a one-minute interval, identifying the most prevalent emotion. The robot’s emotion regulation strategies, such as playing a song or performing a dance, are activated only when negative emotions are predominant within that time frame.
- Speech emotion detection revealed happiness, surprise, and neutral as predominant emotions. Negative emotions (e.g., anger) were rare and context-dependent, primarily occurring as reactions to the robot’s dancing, but related to surprise and excitement rather than anger.

### 4.3. Practical Implications of the Observational Data

The statistical analyses conducted in this study provide valuable insights into the emotional responses elicited during child–robot interaction (CRI). These findings have significant practical implications for the development of emotionally adaptive robotic systems and their application in pediatric contexts.

#### 4.3.1. Implications of *p*-Values

The *p*-values derived from the statistical tests highlight several key differences in emotional responses:

- Significant Anxiety and Fear: The *p*-values for anxiety (p=0.03) and fear (p=0.04) indicate that children experienced these emotions at significantly higher levels than their mothers during CRI. This suggests that children may require additional support when interacting with robots in unfamiliar or potentially stressful situations.
- No Significant Differences in Happiness and Surprise: The lack of significance in happiness (p=0.08) and surprise (p=0.91) between children and mothers indicates that both groups positively engaged with the robot. This reinforces the idea that CRI fosters positive emotional experiences, making it a viable tool for therapeutic or educational interventions.

#### 4.3.2. Implications of the Wilcoxon Signed-Rank Test

The Wilcoxon Signed-Rank Test results (*W*) provide deeper insights into the emotional dynamics between children and their mothers:

- The significant results for anxiety (W=36.0, p=0.03) and fear (W=42.0, p=0.04) highlight the importance of designing robotic behaviors that address these negative emotions. For instance, the robot’s ability to detect and respond to prolonged negative states (e.g., playing music or dancing to alleviate anxiety) proved effective in transitioning children to neutral or positive emotional states.
- The higher guilt scores reported by mothers (W=39.5, p=0.04) suggest that caregivers may experience emotional reactions related to their perceptions of the interaction’s impact on their children. This underscores the need to educate and involve caregivers in the CRI process to ensure a supportive environment.

#### 4.3.3. Implications of Z-Scores

- For anxiety (Z=−2.20) and fear (Z=−2.02), the negative values indicate that these emotions were significantly more pronounced in children. This aligns with developmental psychology theories, which suggest that children at this age are more prone to experiencing heightened anxiety and fear in novel settings. Practical implications include tailoring robotic interventions to gradually acclimate children to the interaction to reduce emotional distress.
- The Z-score for happiness (Z=−1.75) shows a smaller difference between children and mothers, suggesting that both groups benefitted similarly from the robot’s engagement strategies, particularly during positive activities such as dancing and imitation exercises.

#### 4.3.4. Integration into Robotic Design

These findings have direct implications for the design and functionality of emotion-aware robots:

1. Adaptive Emotional Response: Robots should be programmed to detect negative emotions persisting beyond a threshold (e.g., 5 s) and respond with specific strategies such as verbal reassurance, playful gestures, or music to re-engage the child.
2. Parental Inclusion: Designing interventions that include caregivers can help mitigate feelings of guilt or uncertainty, ensuring a collaborative and emotionally supportive environment.
3. Personalized Interaction: The differences in emotional responses suggest that CRI should be personalized to accommodate individual needs, particularly for children who may exhibit heightened anxiety or fear.

The statistical analysis provides robust evidence for the emotional impact of CRI on children and their caregivers. These insights can guide the development of emotion-aware robotic systems that are sensitive to the unique emotional needs of pediatric users and their families. Future work should focus on expanding sample sizes and exploring longitudinal effects to further refine these implications.

### 4.4. Engagement Scores: Gender-Based Analysis

The updated table provides a detailed summary of the engagement scores for 14 children (7 females and 7 males) based on their interactions with the robot. Engagement scores were calculated using a weighted formula that incorporated five factors: proxemics, facial expressions, speech emotion, participation, and mother presence. Each factor was normalized to a 1–5 scale, with weights assigned to reflect its relative importance in determining engagement.

The results show a notable difference between male and female participants. Male participants achieved a higher average engagement score (4.40) compared to females (3.79). This difference is primarily driven by the consistently higher scores for male participants in proxemics and participation, which were heavily weighted in the formula. Male participants were observed to interact more closely with the robot, staying within personal space (≈0.8 m), and demonstrated higher levels of active engagement during activities. Additionally, males scored higher in independence, as none required the presence of a caregiver.

**Figure 10:**
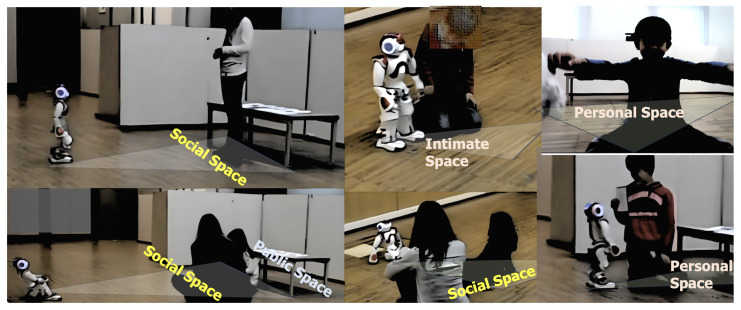
Sample frames showcasing interactions between boys and girls with the NAO robot. The examples highlight their proxemics relative to the robot and instances where mothers participated during specific parts of the session. The images are captured from both the environment camera and the robot’s onboard camera.

In contrast, female participants predominantly maintained social distance from the robot (≈2.0 m), reflected in lower proxemics scores. Their engagement was further influenced by a greater reliance on caregiver presence and a tendency toward neutral facial expressions and speech emotions, which contributed less to the overall score due to their lower weights in the formula. However, some female participants exhibited positive speech emotion and active participation, narrowing the score gap between genders in individual cases. Figure 10 shows examples of boys and girls interacting with the NAO robot, showcasing their proxemics and highlighting specific moments when girls sought the presence of their mother nearby.

It is important to consider that since this study involved only a single session per child, this might have influenced the comfort level, shyness, or confidence of the female participants. In particular, a lack of familiarity with the robot and the environment could have contributed to their preference for maintaining social distance and showing less emotional variability. Longitudinal studies could help reduce these factors over time, as repeated interactions might increase their confidence, trust, and comfort with the robot. Such studies could provide valuable insights into how engagement evolves with familiarity and whether gender-based differences diminish with repeated sessions.

These findings highlight gender-based differences in engagement during child–robot interactions. While males demonstrated higher overall engagement, the results emphasize the influence of behavioral and emotional factors such as proximity and participation. These insights provide a basis for further investigation into tailoring robotic interactions to accommodate individual preferences and needs. However, it is important to note that the sample size is limited, and these results should not be generalized to all children. Future studies with larger cohorts can validate these observations and refine the engagement scoring methodology.

Therefore, we can state that the analysis revealed significant differences in engagement scores between the participants in our study. As demonstrated in Table 7, the gender-based engagement scores highlight differences in sustained attention and responsiveness. Additionally, parental involvement played a crucial role in emotional regulation, particularly for female participants, as parents actively encouraged participation, fostering greater confidence during the interaction. However, despite this increased confidence, female participants maintained a greater social distance from the robot compared to male participants, who engaged in closer personal interactions.

The results analyzed in the context of the formulated hypotheses confirmed H1, demonstrating that adaptive emotional responses significantly increased engagement levels (*p* < 0.05). Gender-based differences (H2) were observed, with male participants exhibiting higher engagement score, as shown in Table 7. Regarding H3, the system maintained stable accuracy across different interaction phases, reinforcing the robustness of multimodal emotion recognition.

### 4.5. Limitations

Due to the small cohort size, and short-term sessions, these findings cannot be generalizable to all children with ADHD or neurotypical peers. However, the data highlight the potential for future studies to explore gender-based differences in proxemics, engagement, and emotional responses during child–robot interactions with a longitudinal study to represent possible patterns between different groups.

## 5. Conclusions and Future Work

The findings of this study demonstrate the transformative potential of emotion-aware technology in real-world applications, particularly in pediatric settings. By integrating affective computing techniques using biophysical data, the system effectively identified and responded to children’s emotional states during child–robot interactions. The results highlight the capability of the NAO robot to elicit positive engagement and emotional regulation across structured activities. For instance, happiness was the dominant emotion in interactive stages like “Icebreaker Dialog” and “Show and Glow”, while neutral expressions prevailed in task-oriented stages such as “Guided Steps” and “Knowledge Exchange”. Minimal occurrences of negative emotions further underscore the robot’s ability to sustain positive and neutral emotional states. Speech emotion analysis revealed a similar progression, with neutral tones dominating the early stages and a significant increase in happy speech during dynamic tasks. Text sentiment analysis corroborated these findings, showing overwhelmingly positive feedback from the children, particularly in engaging stages. These results demonstrate the potential for emotion-aware systems to improve emotional engagement and assist professionals in understanding children’s biophysical and behavioral responses. From a societal perspective, emotion-aware technology can significantly impact healthcare, education, and therapeutic interventions. By enabling robots to detect and adapt to emotional states in real time, this technology promotes emotional well-being, enhances engagement, and fosters personalized experiences, particularly for children with unique emotional or developmental needs.

### Future Work

While the current findings are promising, several areas warrant further investigation:

- Data Expansion and Generalization: Incorporating a more diverse dataset, including additional languages, cultural contexts, and children with neurodevelopmental conditions, will enhance the robustness and applicability of the models.
- Integration of Multimodal Data: Future iterations should incorporate physiological signals such as heart rate or galvanic skin response to provide a more comprehensive emotional profile.
- Adaptive Interventions: The development of more nuanced robot behaviors to address complex emotional states, such as combining verbal reassurances with interactive activities, could further improve emotional regulation.
- Longitudinal Studies: Exploring the long-term impact of emotion-aware robots on children’s emotional and cognitive development will provide deeper insights into their therapeutic potential.

In summary, this study highlights the efficacy of emotion-aware robots in fostering positive interactions and emotional regulation. Continued interdisciplinary research will further bridge the gap between technological innovation and societal impact, creating empathetic systems that enrich human experiences across diverse domains.

## Figures and Tables

**Table 1:** Stages of child–robot interaction with duration and suggested names.

Stage	Duration	Interaction Focus	Task Name
Stage 1	2 min	Verbal communication for introduction and getting to know the child	Icebreaker Dialog
Stage 2	3 min	Gestural communication and imitation exercises	Mirror Me
Stage 3	1 min	Physical interaction, walking hand in hand	Guided Steps
Stage 4	3 min	Teaching the robot about animals using images and verbal explanations	Knowledge Exchange
Stage 5	1 min	Robot demonstration (dance) for visual attention and child feedback	Show and Glow

**Table 2:** Facial expression classification performance on datasets.

Dataset	Accuracy	Precision	Recall	F1 Score
KDEF	89.31%	90%	91%	91%
Real Emotion	88.50%	90%	90%	90%
Merged Dataset	92.00%	94%	94%	94%

**Table 3:** Speech emotion classification performance on individual datasets.

Classifier	Dataset	Precision	Recall	F1 Score	Accuracy
1D CNN	EmoUERJ	89%	84%	84%	86%
MLP	EmoUERJ	79%	81%	79%	77%
DBMM	EmoUERJ	95%	93%	94%	94%
1D CNN	ESD	87%	87%	87%	87%
MLP	ESD	92%	92%	92%	92%
DBMM	ESD	98%	97%	98%	97%

**Table 4:** Speech emotion classification performance on multilingual dataset (EmoUERJ + ESD).

Classifier	Precision	Recall	F1 Score	Accuracy
1D CNN	88%	87%	88%	87%
MLP	94%	94%	94%	94%
DBMM	98%	98%	98%	98%

**Table 5:** Combined results from child–robot interaction across stages.

Stage	Facial Expressions (%)					Speech Emotion (%)		Text Sentiment (%)		
	Happy	Neut	Fear	Sad	Surpr	Neut	Happy	Pos	Neut	Neg
Icebreaker	80	9	5	3	3	75	25	86	7	7
Mirror Me	75	15	5	0	5	75	25	72	14	14
Guided Steps	0	90	5	0	5	93	7	7	86	7
Knowledge Exch	25	70	0	0	5	20	80	93	7	0
Show and Glow	80	20	0	0	0	15	85	100	0	0

**Table 6:** Summary of emotional responses in children and mothers.

Emotion	Children’s Mean (SD)	Mothers’ Mean (SD)	Z-Score	*p*-Value	Wilcoxon Test (W)
Happiness	74.8 (10.3)	85.4 (3.3)	−1.75	0.08	66.0
Surprise	65.3 (17.4)	64.6 (18.9)	−0.12	0.91	45.5
Anxiety	29.2 (25.9)	8.9 (12.5)	−2.20	0.03 *	36.0
Fear	20.3 (19.1)	10.2 (10.0)	−2.02	0.04 *	42.0
Guilt	10.0 (5.0)	18.5 (6.7)	2.02	0.04 *	39.5

Note: Cronbach’s Alpha for children’s emotional responses was α=0.87, indicating excellent internal consistency. For mothers, α=0.68, reflecting acceptable reliability. The Wilcoxon Signed-Rank Test compares paired emotional responses between children and mothers, with W representing the test statistic. * Significant at p<0.05.

**Table 7:** Updated gender-based engagement scores.

Child ID	Gender	Proxemics Distance (m)	Proximity Space	Mother Presence	Engagement Score	Facial Expression	Speech Emotion	Text Feedback
1	Female	1.8	Social	Yes	3.79	Neutral	Neutral	Positive
2	Female	2.0	Social	Yes	3.79	Neutral	Positive	Positive
3	Female	2.0	Social	Yes	3.79	Neutral	Neutral	Positive
4	Female	2.0	Social	Yes	3.79	Neutral	Neutral	Positive
5	Female	2.0	Social	Yes	3.79	Neutral	Neutral	Positive
6	Female	2.0	Social	Yes	3.79	Neutral	Neutral	Positive
7	Female	1.8	Social	Yes	3.79	Positive	Neutral	Positive
8	Male	0.7	Personal	No	4.40	Neutral	Positive	Positive
9	Male	0.7	Personal	No	4.40	Neutral	Neutral	Positive
10	Male	0.7	Personal	No	4.40	Positive	Neutral	Positive
11	Male	0.8	Personal	No	4.40	Positive	Positive	Positive
12	Male	0.8	Personal	No	4.40	Positive	Neutral	Positive
13	Male	0.8	Personal	No	4.40	Positive	Neutral	Positive
14	Male	1.0	Personal	No	4.40	Positive	Neutral	Positive
Average	-	Female: 1.94; Male 0.79	-	-	Female: 3.79; Male: 4.40	-	-	-

## Data Availability

Data are contained within the article.
